# Enantioselective transacetylation of (*R,S*)-*β*-citronellol by propanol rinsed immobilized *Rhizomucor miehei *lipase

**DOI:** 10.1186/1752-153X-1-10

**Published:** 2007-04-18

**Authors:** Abir B Majumder, Shweta Shah, Munishwar N Gupta

**Affiliations:** 1 Chemistry Department, Indian Institute of Technology Delhi, Hauz Khas, New Delhi 110016, India

## Abstract

**Background:**

Use of enzymes in low water media is now widely used for synthesis and kinetic resolution of organic compounds. The frequently used enzyme form is the freeze-dried powders. It has been shown earlier that removal of water molecules from enzyme by rinsing with n-propanol gives preparation (PREP) which show higher activity in low water media. The present work evaluates PREP of the lipase (from *Rhizomucor miehei*) for kinetic resolution of (*R,S*)-*β*-citronellol. The acylating agent was vinyl acetate and the reaction was carried out in solvent free media.

**Results:**

The PREP, with 0.75% (v/v, reaction media) water, was indeed found to be more efficient and gave 95% conversion to the ester. Using this PREP, with no added water, 90% ee for (*R*)-(+)-*β*-citronellyl acetate at 45% conversion (E = 42) was obtained in 4 h. The control with freeze-dried enzyme, with zero water content, gave 78% ee at 30% conversion (E = 13). FT-IR analysis showed that PREP had retained the α-helical content of the enzyme. On the other hand, freeze-dried enzyme showed considerable loss in the α-helical content.

**Conclusion:**

The results show that PREP may be a superior biocatalyst for enantioselective conversion by enzymes in low-water media.

## Background

Use of enzymes in non-aqueous media for organic transformations is now a well-established approach [1,2]. It has been shown that propanol rinsed enzyme preparations, (PREPs) show higher activity than the freeze-dried enzyme preparations in such low water media [3,4]. This higher activity of PREPs is believed to be due to the fact that unlike freeze drying, removal of excess water by rinsing with propanol does not cause reversible structural changes in the enzyme molecule [5]. However, there is hardly any information regarding enantioselectivity of PREPs as compared to the freeze-dried preparations. The present work makes this comparison with the enantioselective synthesis of (*R*)-*β*-citronellyl acetate using immobilized *Rhizomucor miehei *lipase, Lipozyme^® ^RM IM. Lipozyme^® ^RM IM is a commercially available lipase which has been frequently used for transesterification reactions [6]. Generally, transesterification reactions are carried out in organic solvents containing low amount of water. One underexploited approach is the use of solvent free media, i.e. using reactant(s) as such as reaction media. This approach offers several advantages [7]. These advantages include: (a) solvents cost are eliminated (b) solvent free system offer greater safety (c) increased reactant concentration and (d) facile product separation. The present work was carried out in solvent free media.

In order to evaluate the performance of PREP of the lipase for enantioselectivity, it was decided to work (instead of a model reaction) with a reaction in which both the substrate and the product are useful as pure enantiomers. The reaction was transacetylation of (*R,S*)-*β*-citronellol by vinyl acetate. Citronellyl acetate is an important terpene ester which finds extensive applications in food, cosmetics, pharmaceutical industries [8]. Wang and Linko [9] have reported that both (*R*)-*β*-citronellyl acetate and (*S*)-*β*-citronellyl acetate have different flavors. These workers carried out enantioselective esterification of citronellyl butyrate with somewhat limited success [9]. Citronellols are also used as flavors. Both (*R*)-*β*-citronellol and (*S*)-*β*-citronellol have different flavors. Hence an enantiomerically pure alcohol left behind after enantioselective conversion would also be valuable. Lipases have been extensively used for enantioselective synthesis of esters from alcohols. In general, lipases show poor enantioselectivity towards primary alcohols. This is especially so for primary alcohols wherever the chiral carbon is not adjacent to the alcoholic group. Citronellol is such a primary alcohol with a "remote chiral center" [10,11]. Thus enzymatic resolution of citronellol via the enantioselective synthesis of citronellyl acetate has been a difficult job [12]. As pointed out by Oda et al, [10] although Cambou and Klibanov [12] reported the kinetic resolution of (*R,S*)-citronellol via transesterification with hog liver carboxylesterase, high cost and the instability of the enzyme makes that process impractical. Thus, the biotransformation of citronellol to the ester is a challenging system to evaluate comparative efficiency of PREP over frequently used freeze-dried powders in enantioselective reactions.

## Results and discussion

As a first step, the conditions for the conversion of citronellol to citronellyl acetate with Lipozyme^® ^RM IM were optimized. Vinyl acetate was used for transesterification since it is known to be an efficient reagent for transesterification [1]. The reason for its efficiency is the instability of vinyl alcohol which immediately isomerizes to acetaldehyde. This drives the reaction forward.

After optimization in terms of molar ratio of vinyl acetate to racemic-*β*-citronellol, and water content, it was found that freeze-dried preparation of Lipozyme^®^RM IM showed 90% conversion to (*R,S*)-*β*-citronellyl acetate by using 1:6 molar excess of vinyl acetate with 0.3% (v/v, reaction medium) water content in 8 h at 40°C. The PREP was indeed found to be more efficient. The % conversion to racemic-*β*-citronellyl acetate in 4 h at 40°C increased from 80% to 95% when 0.75% water (v/v) was added to the reaction medium (data not shown in the form of figure/table). Hence it was thought worthwhile to evaluate the PREP for enantioselective conversion in this case.

**Figure 1:**
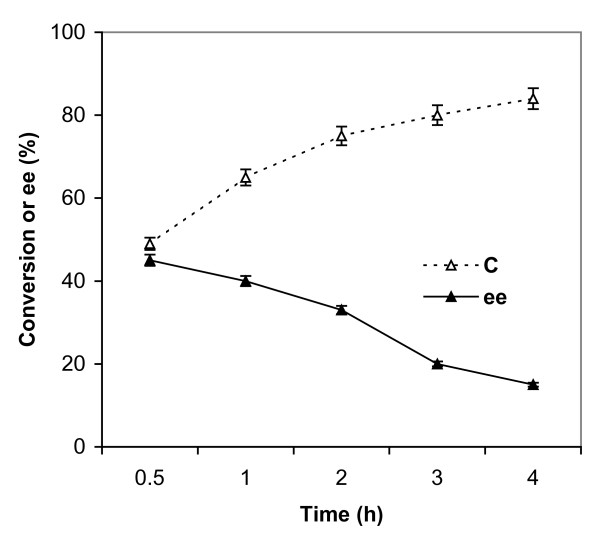
**Changes in the percentage conversion (C) and ee (%) values for freeze-dried lipase with time**. Citronellol and vinyl acetate were taken in 1:6 (mol/mol) ratio in screw capped vials with freeze-dried lipase (10% w/w, reactants) containing 0.3% (v/v) water. The reaction mixtures were incubated at 40°C. Each point represents the outcome of a pair of readings and the deviations were less than 3%.

Fig. 1 shows that enantioselective conversion of (*R,S*)-*β*-citronellol to (*R*)-citronellyl acetate with freeze dried lipase at 40°C. At 49% conversion, to (*R*)-citronellyl acetate, enantiomeric excess (ee) was 45% in 0.5 h. The corresponding E value was 4.

**Figure 2:**
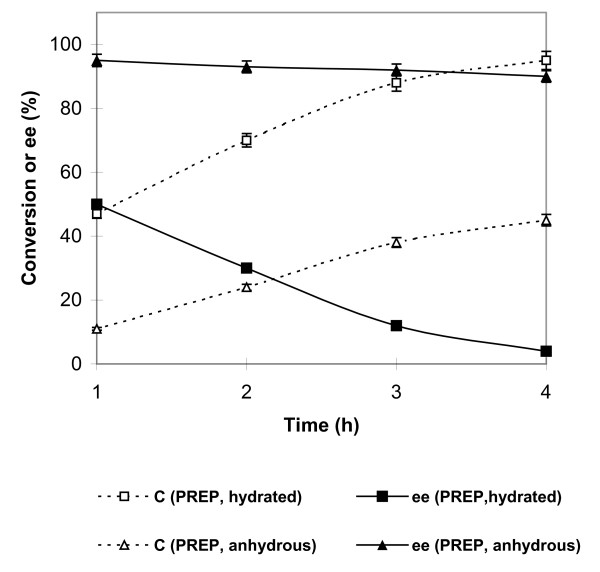
**Changes in the percentage conversion (C) and ee (%) values for PREPs with time**. Citronellol and vinyl acetate were taken in the 1:6 (mol/mol) ratio in the screw-capped vials. The PREPs, both anhydrous and hydrated by 0.75% water (v/v), (10%, w/w reactants) were used. The vials were incubated at 30°C at 200 rpm. Each point represents the outcome of a pair of readings, which differed by less than 3%, with many points showing errors which are smaller than the symbols.

Fig. 2 shows the performance of PREPs with 0.75% (v/v) water which was found optimum for conversion and without any water addition at a lower temperature of 30°C. In this case, lower temperature of 30°C was used for improving enantioselectivity. The improvement in enantioselectivity with lowering of the temperature has been reported by earlier workers [13]. In this case, when no water was added to the reaction medium, 90% ee for (*R*)-*β*-citronellyl acetate at 45% coversion (E = 42) was obtained in 4 h. Decrease in water content has been reported to improve enantioselectivity by others as well [14]. It may be added that both lowering of the temperature and reduction in water content leads to decrease in conformational flexibility. This decrease in conformational flexibility is supposed to be the cause of improved enantioselectivity [1]. A control with freeze dried enzyme operating at 30°C and without any added water gave 78% ee at 38% conversion (E = 13) only and with 0.3% v/v water (at 30°C) the ee value was 70% at 48% conversion resulting an E = 10 (data not shown as a figure or a table).

Some earlier efforts have also been carried out for the kinetic resolution of racemic citronellol. Cambou and Klibanov (1984) [12] had tried transesterification of citronellol with methyl propionate by hog liver carboxyl esterase lipase in aqueous-organic biphasic reaction media and they got 98% optically pure ester. Later on Oda et al (1999) [10], using a system containing acetyl Co-A, in a biphasic reactor system, had an improved enantiomeric excess of 98% at 40% conversion. However, in both of the above approaches, the biocatalysts had shown the (*S*)-enantioselectivity. Wang and Linko (1995) [9] had done the esterification of citronellol with butyric acid catalyzed by lipases from *Candida rugosa*, *R. japonicus*, *P. fluorescence *and showed the (*R*)-enantiopreference. Recently, Sankaranarayanan et al (2003) [15] have done kinetic resolution with porcine pancreatic lipase as a part of a synthesis. They had shown (*R*)-enantiomeric excess of 93% of citronellyl acetate at 30% conversion with vinyl acetate in presence of solvent (hexane) in 24 h.

The improved performance of PREP in the present instance is in agreement with earlier observations that such lipase preparations show greater enantioselectivity (as compared to the lyophilized powder) in dry hexane [16] and ionic liquids [4].

In order to understand the structural basis for better performance of PREP, both the freeze-dried enzyme and the PREP were examined by FT-IR. A considerable loss in α-helix content of the untreated Lipozyme was observed after freeze-drying. The α-helical content was 21% in the case of the untreated enzyme and 5% for the freeze-dried enzyme (Table 1, see additional file 1). This is in agreement with earlier studies which investigated the structural consequences of lyophilization [4]. The PREP, on the other hand, showed 28% α-helix which was even marginally better than untreated ("directly from the bottle") sample. It is interesting to note that the X-ray diffraction had shown that α-helix content of this lipase is 28% [16]. It is not known what procedures were employed by the manufacturer for preparing this enzyme. The structural changes brought by the freeze-drying are reversible. Such preparations, used directly as such, show low activity under low water conditions [5]. Crystals used for X-ray diffraction are obtained from aqueous solutions. Hence, X-ray diffraction is expected to give the correct value for the α-helical content in the native structure of the enzyme. The agreement with α-helical content values obtained by the X-ray diffraction [17] and by FT-IR of PREP in the present study shows that propanol rinsing (as compared to freeze-drying) led to the better retention of enzyme conformation. This explains its higher activity in organic solvent.

**Table 1:** FT-IR analysis (Deconvolved by OMNIC 2.1 software) of the Enzyme formulations

Peak positions (cm^-1^)	Untreated (RML) (Peak area)	FD (RML) (Peak area)	PREP (RML) (Peak area)
1609	0.057	0.036	0.012
1618	-	0.015	0.008
1628 ± 1	-	0.018	0.007
1637 ± 3 (β-sheet)	0.017	0.011	0.049
1650 ± 5 (α-helix)	0.026	0.008	0.045
1665 ± 5 (coil)	0.014	0.006	0.0101
1678 ± 2 (β-turn)	-	0.011	0.0139
1688 ± 2 (β-sheet)	0.012	0.019	0.0206
1700	-	-	0.0112

* Untreated : directly from the bottle

## Conclusion

To sum up, high conversion of 95% for obtaining (*R,S*)-*β*-citronellyl acetate in the shortest possible time of 4 h could be obtained using PREP. For obtaining (*R*)-(+)-*β*-citronellyl acetate with the highest E of 42 again was possible by PREP under anhydrous condition. The conversion yield of the unreacted citronellol was 55% with a 70% enantiomeric excess of (*S*)-(-)-*β*-citronellol. It is not often appreciated that enzyme catalyzed reaction in organic medium tend to be slow [7]. Quite often these reactions are slower than the same reaction carried out by chemical catalysis [7]. Hence experience with enzyme formulations which show higher reaction rates is important in the context of organic transformations. The present work shows that PREP constitutes a promising biocatalyst formulation for carrying out kinetic resolution with high enantioselectivity.

## Experimental

*Rhizomucor miehei *lipase immobilized on macroporous anion exchange resin (Lipozyme^®^RM IM) was a kind gift from Dr. J.S. Rao, Novozymes, Bangalore, India. Racemic citronellol was a gift by Gogia chemical, New Delhi, India. S-(-)-citronellol and vinyl acetate (>99%, GC grade) were purchased from Merck, Honenebrunn, Germany.

### Freeze-dried enzyme preparation

Lipozyme was suspended in 0.5 ml of 20 mM phosphate buffer, pH 7 for 1 h at 25°C. This was frozen at -20°C and lyophilized for 24 h.

### Propanol-rinsed enzyme preparation (PREP)

The Lipozyme was suspended in 0.5 ml of 20 mM phosphate buffer, pH 7, for 1 h at 25°C. The mixture was cooled to 4°C. The supernatant was carefully pipetted out and the lipase was rinsed thrice with ice-cold n-propanol at 4°C (containing varied amounts of water %, v/v) followed by washing with ice-cold vinyl acetate (with the same corresponding water concentrations) at 4°C.

### Transesterification of citronellol

Citronellol and vinyl acetate were taken in the ratio of 1:6 (mol/mol) in a screw capped vial followed by addition of different preparations of Lipozyme^® ^RM IM (10% w/w, reactants). The reaction mixtures were incubated at varied temperatures with a constant shaking at 200 rpm. Aliquots were taken out at various time intervals and the citronellyl acetate formed was analyzed by Gas Chromatography (Agilent 6890 N chromatograph with a flame ionization detector). The capillary column used was EQUITY™^-5 ^(30 m × .32 mm × 0.25 μm film thickness) from Supelco. N_2 _was used as the carrier gas at a constant pressure of 4 Kg/cm^2^. The column oven temperature was programmed from 150 to 250°C (at the rate of 10°C/min) with injector and detector temperatures at 240 and 250°C, respectively.

### Determination of ee & E

Reaction aliquots at different time intervals were taken and the ee values were determined by HPLC with a chiral stationary phase (Chiracel OD RH, from Daicel, Japan). The eluent was a mixture of acetonitrile and 0.1 M phosphate buffer (pH 2.5) in (9:1, v/v) ratio. Analysis was done at 210 nm with a flow rate of 0.1 ml/min. The peaks were identified by running optically pure (*R*)-,(*S*)-citronellols and their acetates taken as standards. From the ee values (calculated from the peak areas of the enantiomers), the enantioselectivity (E) was calculated by Chen's equation as described earlier [4].

### FT-IR analysis of enzyme preparations

The spectra was recorded on Nicolet™ FT-IR 6700 spectrophotometer with Smart Miracle™ ATR accessory having zinc selenide crystal. The data collection and deconvoluation were done by using OMNIC 2.1 Software. Different formulations of Lipozyme, freeze-dried and PREP (anhydrous) were taken directly on the zinc selenide crystal plate and an average of 200 scan were taken with a resolution of 2 cm^-1^. The spectra were analyzed over 1600 cm^-1 ^to 1700 cm^-1 ^(Amide-I band).

## Supplementary Material

Additional file 1 The FT-IR spectra of different formulations of Lipozyme^®^RM IM and the secondary structure of the enzyme formulations based on FT-IR analysis are given. Click here for file
