# Supplementary material for: Enantioselective transacetylation of (R,S)-β-citronellol by propanol rinsed immobilized Rhizomucor miehei lipase
Source: Chem Cent J. 2007 Apr 18;1:10. doi: 10.1186/1752-153X-1-10 (PMC1994055; doi:10.1186/1752-153X-1-10)
Supplement: Additional file 1 — The FT-IR spectra of different formulations of Lipozyme®RM IM and the secondary structure of the enzyme formulations based on FT-IR analysis are given. [file 1752-153X-1-10-S1.doc]

**Additional data file:**

Wave number (cm^-1^)

**Figure 1.** Deconvolved amide I bands for different formulations of Lipozyme: The spectra were recorded as described in the experimental section. The composite spectrum describes the spectra of the untreated (red), the freeze-dried (purple) and that of anhydrous PREP (blue) of Lipozyme ® RM IM.

**Table 2. Secondary structure of the enzyme formulations based on FTIR analysis** :

| Enzyme formulations (RML) | α-helix | β-sheet | Others |
| --- | --- | --- | --- |
| Untreated | 21 % | 13 % | 56 % |
| Freeze-Dried | 5 % | 19 % | 86 % |
| PREP | 28 % | 44 % | 28 % |
